# Supplementary material for: Solvent-Free and Efficient One-Pot Strategy for Synthesis of the Triazine-Heterocycle Azacyanines
Source: Materials (Basel). 2022 Apr 2;15(7):2619. doi: 10.3390/ma15072619 (PMC9000731; doi:10.3390/ma15072619)
Supplement: Supplementary file 1 [file materials-15-02619-s001.zip › materials-1616070-supplementary.pdf]

Supplementary Information

Solvent-free and efficient one-pot strategy for synthesis of the triazine-heterocycle azacyanines

Xianwu Jiang [1], Zhuodong Sun [2] and Yu Wang [1,*]

[1]School of Chemical Engineering, East China University of Science and Technology, Shanghai, 200237, China.

[2]M.S, School of Chemistry and Molecular Engineering, East China Normal University, Shanghai 200241, China.

[1*]School of Chemical Engineering, East China University of Science and Technology, Shanghai, 200237, China.

1. The mechanism of the reaction to prepare of azacyanines

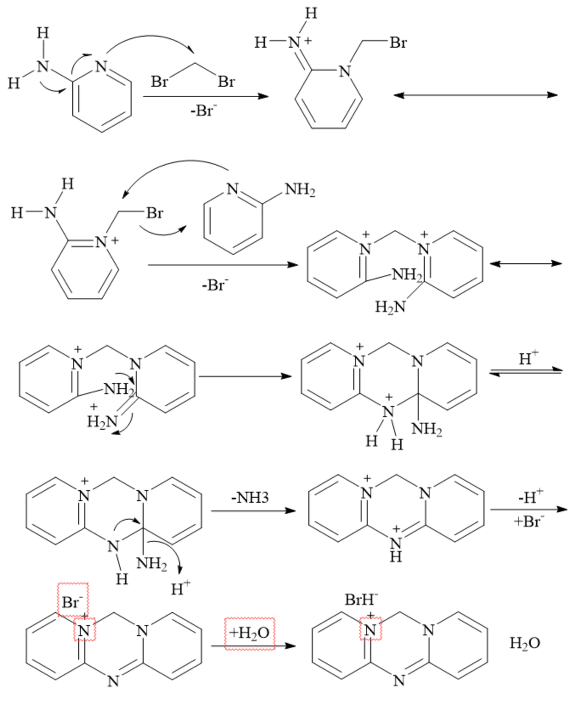

Figure S1. The mechanism of the reaction between 2-aminopyridine and dibromomethane.

The mechanism of the reaction is that the bromo group on dibromomethane was nucleophilic attack on the amino group of pyridine, namely one molar ratio of dibromomethane was reacted with 2 molar ratio aminopyridine through intramolecular cyclization reaction to sequentially synthesis of final structure, triazine-heterocycle azacyanines.

2. Single crystal diffraction results of Azacyanine 1.

Table S1. Crystal data and structure refinement for Azacyanine 1

Program	Value
Empirical formula	$C_{44}H_{40}Br_4N_{12}O_4$
Formula weight	1120.48 g/mol
Temperature	295±2 K
Wavelength	0.071073 nm
Crystal system	monoclinic system
Space group	P21
Unit cell dimensions	a=5.495±2 Å α=90°<br>b=11.939±4 Å β=95.370±6°<br>c=17.717±6 Å γ=90°
Volume	1157.2±7 $Å^3$
Z	1
Calculated density	1.608 mg/m^3
Absorption coefficient	3.534 mm^{-1}
F(000)	560
Limiting indices	$-7 \leq h \leq 4$, $-15 \leq k \leq 15$, $-22 \leq 1 \leq 22$
Theta range for data collection	$2.06° \leq θ \leq 27.50°$
Completeness to theta	97.3%
Goodness-of-fit	2.756
Final R indices	R_1=0.1862
[I>2sigma(I)]	wR_2=0.5380
R indices	R=0.2011
(all data)	wR_2=0.5512
Largest diff. peak and hole	4.363 $Å^{-3}$、-1.818e $Å^{-3}$

The description of the crystal data information such as chemical formula, molecular weight, crystal system and space group, temperature, unit cell parameters, model of the diffractometer, type of the absorption correction, number of independent and

observed reflections, number of refined parameters show in Table S1.

3. The unit cell of Azacyanine 1

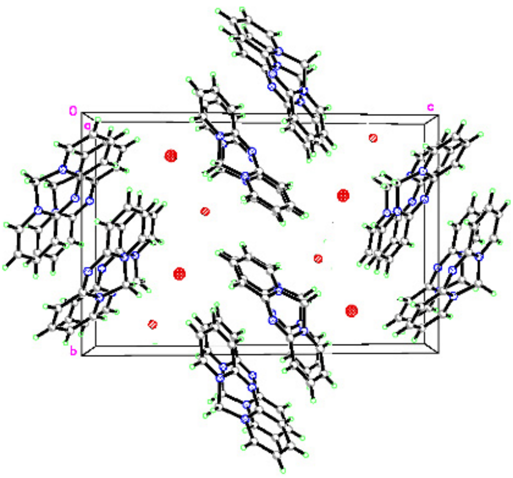

Figure S2. Crystal packing diagram of Azacyanine 1

Figure S2 presents the unit cell of Azacyanine 1. It is the crystal cell accumulation diagram of the molecule along the c axis.
